# Draft genome sequence of *Escherichia* sp. F1, an iron-reducing bacterium isolated from chlorinated hydrocarbon-contaminated soil

**DOI:** 10.1128/mra.00068-25

**Published:** 2025-06-18

**Authors:** Zhineng Wu, Linhao Kang, Hanyu Niu, Yaduo Yang, Xiaodong Ma

**Affiliations:** 1 School of Energy and Environmental Engineering, Hebei University of Technology 12606, Tianjin, China; California State University San Marcos, San Marcos, California, USA

**Keywords:** *Escherichia*, iron-reducing bacterium, soil, degradation

## Abstract

We report the draft genome sequence of an iron-reducing bacterium, *Escherichia* sp. F1. The genome of strain F1 comprises 4,989,064 bp with a GC content of 50.59%, encoding 4,771 genes. Notably, genes associated with Fe(III) reduction and (S)-2-haloacid dehalogenase activity were identified, highlighting their potential for environmental remediation.

## ANNOUNCEMENT

The genomic sequencing of *Escherichia* sp. F1 was conducted to investigate its genetic potential for Fe(III) reduction and organohalide degradation (1–4).

Soil samples (1 kg) were collected from a chlorinated hydrocarbon-contaminated site in Zhongshan City, Guangdong Province, China (113°14′38″E, 22°33′37″N) using the plum blossom sampling method. Strain F1 was isolated under anaerobic conditions: 10 g of soil was suspended in 50 mL of sterilized saline (0.9%), incubated anaerobically for 3 hours, and 2 mL of the suspension was transferred to 20 mL ferric citrate medium. The medium composition included 3 g/L FeC_6_H_5_O_7_, 1 g/L NH_4_Cl, 0.07 g/L CaCl_2_·2H_2_O, 0.6 g/L MgSO_4_·7H_2_O, 0.722 g/L K_2_HPO_4_·3H_2_O, 0.25 g/L KH_2_PO_4_, 1 g/L C_6_H_12_O_6_, supplemented with 1 mL/L of trace element stock solution and 0.077 g/L C_4_H_10_O_2_S_2_ (5). After daily agitation at 30°C, the medium transitioned from brownish-yellow to colorless, indicating Fe(III) reduction. Three sequential enrichments were performed, followed by streaking on LB agar and incubating at 30°C for 48 hours. A dominant colony was purified through five subcultures. The resulting pure strain of iron-reducing bacterium was isolated and designated as strain F1.

Strain F1 was cultured in liquid LB medium at 30°C for 18 hours until it reached the log phase. Genomic DNA was extracted using the QIAGEN Genomic-tip 20/G kit, and sequencing was conducted by Biomarker Technology Co. (Beijing, China). The DNA was quantified via a NanoDrop 2000 spectrophotometer (Thermo Fisher Scientific) and Qubit TM3 fluorometer (Thermo Fisher Scientific). Library preparation involved shearing 15 µg of DNA with g-TUBE (Covaris) according to the manufacturer’s protocol to achieve a target fragment size of ~15 kb, as required for PacBio long-read sequencing. Small fragments (<1 kb) were removed using AMPure PB Beads to enrich larger fragments and improve library quality. The SMRTbell library was constructed following the instructions of the SMRTbell Express Template Preparation Kit 2.0 (PacBio, Cat No. 100-938-900) and sequenced on the PacBio SMRT Sequel II platform (6). Sequencing reads were quality-checked using PacBio SMRT Link (v10.2), achieving an average quality score >Q20. The PacBio SMRT platform produced a total of 9,304,436 reads (N_50_, 7,951 bp). Genome assembly was carried out using Hifiasm v0.16.1 (7), circularized with Circlator v1.5.5 (8), and polished with Pilon v1.22 (9). The summary of the assembly and annotation statistics for strain F1 is presented in Table 1.

**TABLE 1:** Summary of the assembly and annotation statistics for *Escherichia* sp. F1

Parameter	Value or result
Genetic element	Chromosome
Genome size (bp)	4,989,604
GC content (%)	50.59
No. of total genes	4,771
No. of tRNAs	88
No. of rRNAs	7
GenBank accession number	CP177202

Functional annotation was performed using the NCBI Prokaryotic Genome Annotation Pipeline (PGAP v6.9) (10). The annotation identified 53 genes related to Fe(III) reduction and two genes associated with (S)-2-haloacid dehalogenase activity (2). Phylogenetic analysis of the 16S rRNA gene was conducted after genome annotation. The 16S rRNA sequence was compared to the NCBI nucleotide database using BLAST. A phylogenetic tree was constructed using MEGA 11.0 (11) software, employing the Neighbor-Joining method. Strain F1 revealed 99.71% similarity to *Escherichia* coli strain 160-c pink, confirming its classification as *Escherichia* sp. These findings underscore the potential of strain F1 in Fe(III) reduction and organohalide degradation, although biochemical validation is necessary.

## Data Availability

The draft genome sequence of *Escherichia* sp. F1 has been deposited in GenBank under the accession number CP177202, BioProject number PRJNA1201813, and BioSample number SAMN45944655. The raw sequence reads are available under Sequence Read Archive accession number SRR32075555 (PacBio).
